# Should Transformation Products Change the Way We Manage Chemicals?

**DOI:** 10.1021/acs.est.4c00125

**Published:** 2024-04-24

**Authors:** Daniel Zahn, Hans Peter H. Arp, Kathrin Fenner, Anett Georgi, Jasmin Hafner, Sarah E. Hale, Juliane Hollender, Thomas Letzel, Emma L. Schymanski, Gabriel Sigmund, Thorsten Reemtsma

**Affiliations:** † Helmholtz Centre for Environmental Research - UFZ, Permoserstrasse 15, 04318 Leipzig, Germany; ‡ Norwegian Geotechnical Institute (NGI), P.O. Box 3930, Ullevål Stadion, 0806 Oslo, Norway; § Department of Chemistry, Norwegian University of Science and Technology (NTNU), N-7491 Trondheim, Norway; ∥ Swiss Federal Institute of Aquatic Science and Technology (Eawag), 8600 Dübendorf, Zürich, Switzerland; ⊥ Department of Chemistry, University of Zürich, 8057 Zürich, Switzerland; # TZW: DVGW Water Technology Center, Karlsruher Str. 84, 76139 Karlsruhe, Germany; ∇ ETH Zurich, Institute of Biogeochemistry and Pollutant Dynamics, Zürich 8092, Switzerland; ○ AFIN-TS GmbH (Analytisches Forschungsinstitut für Non-Target Screening), Am Mittleren Moos 48, 86167 Augsburg, Germany; ● Luxembourg Centre for Systems Biomedicine (LCSB), University of Luxembourg, 6 avenue du Swing, L-4367 Belvaux, Luxembourg; △ Environmental Technology, Wageningen University & Research, 6700 AA Wageningen, The Netherlands; ▲ University of Leipzig, Linnéstrasse 3, 04103 Leipzig, Germany

**Keywords:** Water management, emerging contaminants, nontarget screening, advanced oxidation processes, risk assessment

## Abstract

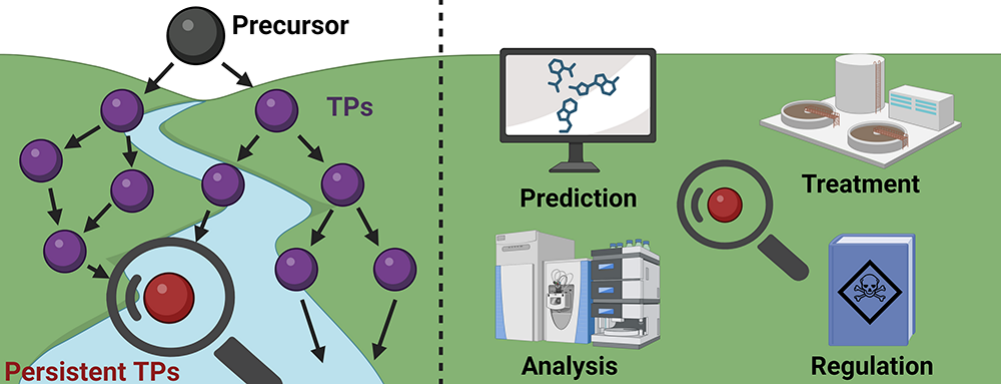

When chemical pollutants enter the environment, they can undergo diverse transformation processes, forming a wide range of transformation products (TPs), some of them benign and others more harmful than their precursors. To date, the majority of TPs remain largely unrecognized and unregulated, particularly as TPs are generally not part of routine chemical risk or hazard assessment. Since many TPs formed from oxidative processes are more polar than their precursors, they may be especially relevant in the context of persistent, mobile, and toxic (PMT) and very persistent and very mobile (vPvM) substances, which are two new hazard classes that have recently been established on a European level. We highlight herein that as a result, TPs deserve more attention in research, chemicals regulation, and chemicals management. This perspective summarizes the main challenges preventing a better integration of TPs in these areas: (1) the lack of reliable high-throughput TP identification methods, (2) uncertainties in TP prediction, (3) inadequately considered TP formation during (advanced) water treatment, and (4) insufficient integration and harmonization of TPs in most regulatory frameworks. A way forward to tackle these challenges and integrate TPs into chemical management is proposed.

## Abstract 2

Advancements in prediction and analysis of transformation products are required to facilitate better assessment and management of chemicals.

## Introduction

Persistence has been a defining property used in chemicals management since the early 1960s, when regulations were introduced that surfactants put on the market had to exhibit primary degradation in the environment.^1^ Persistence is a key component of the Stockholm Convention on Persistent Organic Pollutants^2^ and more recently the European Union’s Chemical Strategy for Sustainability toward a toxic-free environment.^3^ Less attention has been given to the transformation products (TPs) formed from nonpersistent chemicals, despite the fact that many examples of harmful or/and persistent TPs exist: oxidation of the tire antioxidant *N*-(1,3-dimethylbutyl)-*N*′-phenyl-*p*-phenylenediamine (6-PPD) leads to the formation of 6-PPD quinone, which is suspected to cause acute mortality events of coho salmon in the U.S. Pacific Northwest,^4^ the preservative bronopol (2-bromo-2-nitro-1,3-propanediol) transforms to toxic 2-bromo-2-nitroethanol and bromonitromethane in aquatic environments,^5^ and the fluorinated telomer alcohol 8:2 FTOH is one of many precursors of the widely regulated persistent (P), mobile (M), bioaccumlative (B), and toxic (T) perfluorooctanoic acid.^6^ Consequently, both hazard and risk assessment of a chemical are incomplete if its TPs are not considered. So far, discovery of harmful TPs is often incidental and delayed, occurring years or even decades after the introduction of the precursor chemical onto the market. Thus, a more systematic assessment of TPs is essential to evaluate their risk early enough to act and not just react.^7,8^

**Figure 1:**
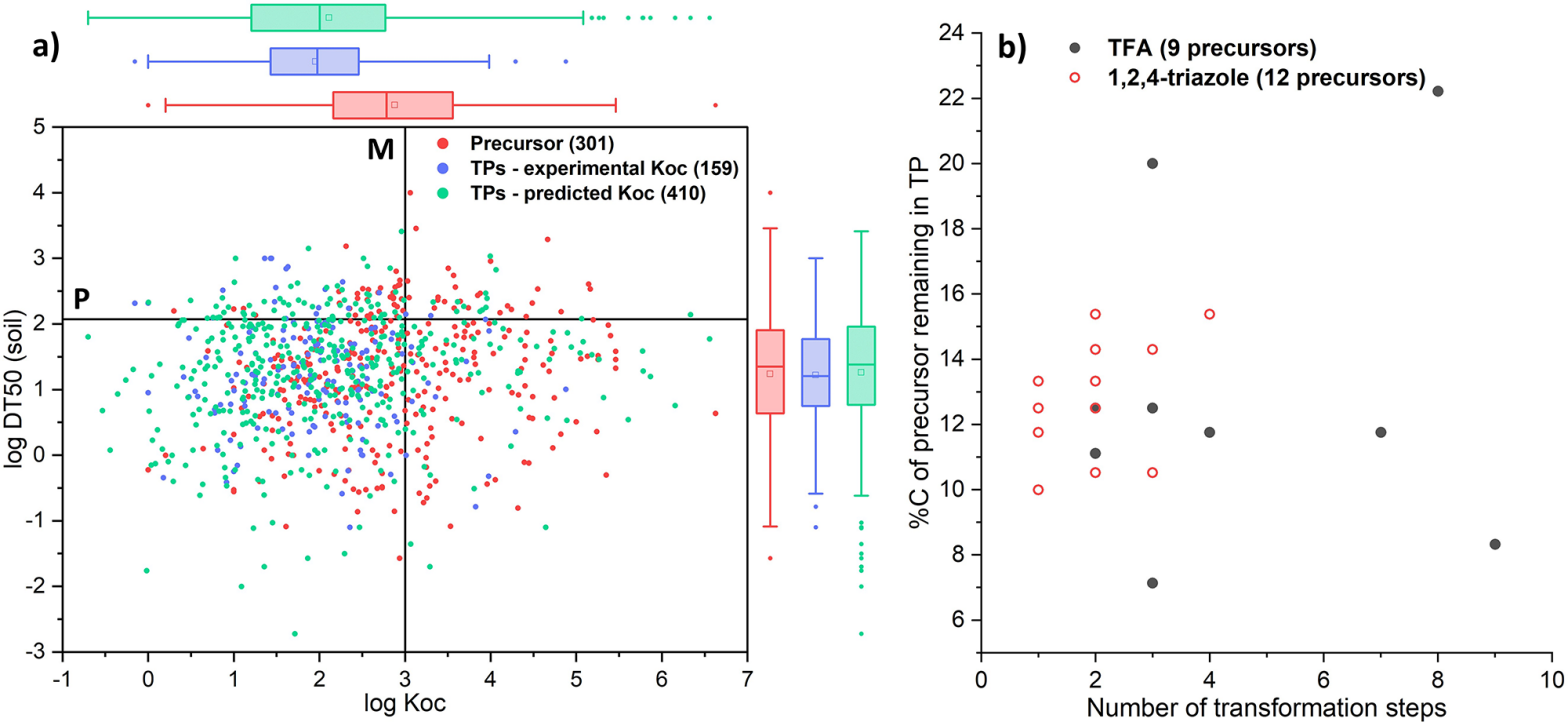
a) Scatterplot of log Koc and median DT50 values for pesticides and pesticide TPs. All DT50 values were taken from enviPath (www.envipath.org; Package: EAWAG-SOIL). High-quality Koc data for all pesticides and 159 TPs were extracted from the pesticide properties database (http://sitem.herts.ac.uk/aeru/ppdb/), while Koc values for an additional 410 TPs were calculated with KOCWIN, EPI Suite.^11^ (b) Scatter plot of the lowest number of predicted or observed transformation steps required to reach the persistent TPs TFA (www.envipath.org; Package: TP predictions TFA (BBD, top 50)) or 1,2,4-triazole (Package: EAWAG-SOIL) and the percentage of carbon of the precursor still remaining in the TPs for their precursor compounds among the 317 pesticides in (a).

Common arguments why TPs are often neglected in chemicals management are that (I) they are often more oxidized than their precursor and thus tend to be more polar (Figure 1a) and less bioaccumulative, limiting exposure through the food web, (II) in many cases the toxicologically active site is degraded, leading to TPs that are less toxic than their precursors, and (III) low transformation yields and several parallel transformation routes imply lower environmental concentrations. However, scenarios exist where these three arguments do not apply. This is the case (I) if TPs are persistent and mobile, such that they accumulate in drinking water resources and represent a chronic source of exposure if continuously emitted,^9^ (II) if degradation products are more toxic than their precursors as presented in the examples above, and (III) if there is a persistent substructure within the molecule, in which case the ultimate yield of this substructure from several different precursors can be high, such as for trifluoroacetic acid (TFA)^10^ and 1,2,4-triazole (Figure 1b).

Consequently, neglecting TPs may challenge a circular economy: the buildup of harmful TPs in products and wastes may complicate their recycling. Persistent and mobile TPs can spread in the water cycle, preventing circular water use. Due to insufficient assessment, harmful TPs formed from replacement products lead to regrettable substitution that may only be recognized after years of chemical use. This Perspective addresses the main challenges for a better assessment and integration of TPs into various aspects of chemicals management and proposes a way forward to eventually overcome them.

## An Analytical Challenge?

Chemical analysis is essential to investigate the presumably large number of TPs formed in the environment and technical systems, but the comprehensive analysis of TPs faces several challenges. A major one is that the generally increased polarity of TPs compared to their precursors results in a larger fraction falling outside the polarity window of widely used sample preparation techniques as well as reversed phase liquid chromatographic (RPLC) and gas chromatographic separation methods. They may thus elute in the void volume of a sample run, which renders detection with these methods difficult.^12,13^ Consequently, alternative enrichment (e.g., freeze-drying,^14^ evaporative concentration,^15,16^ and multilayer solid phase extraction^16^) and separation methods (e.g., hydrophilic interaction liquid chromatography,^17^ mixed mode chromatography,^14^ capillary electrophoresis,^18^ ion chromatography,^19^ and polar column supercritical fluid chromatography^20,21^) are required to extend the analytical window toward higher polarity chemicals and successfully retain the more polar TPs. While the applications of these methods are steadily increasing, they are not yet widespread enough to adequately complement RP-HPLC and have to be used in addition to them, requiring additional analysis and data processing time. At this stage, there is no one generic method that can reliably cover the entire range of polar chemicals (e.g., pronounced matrix effects are observed for freeze-drying or evaporative concentration, while ion exchange chromatography is limited to charged chemicals), hampering the analysis of the most polar TPs.

Since TPs are rarely available as analytical standards, they are typically investigated using nontarget approaches, involving either suspect screening (for documented or predicted TPs) or full nontarget screening workflows.^22^ These workflows rely on mass spectral libraries (up to a Level 2a identification confidence^23^) or compound databases combined with *in silico* fragmentation approaches (lower confidence identification, typically Level 3). A concerted effort by the environmental community including the NORMAN Network,^24^ FOR-IDENT,^25^ MassBank (www.massbank.eu), CompTox,^26^ and PubChem^27^ has seen a marked increase in openly available TP information in recent years. Yet TPs account for only a tiny fraction of the entries in these databases (3.6% and 0.1% in MassBank EU or MassBank of North America (https://mona.fiehnlab.ucdavis.edu/), respectively). When novel TPs are identified through nontarget screening in complex samples, the identification of precursors can be challenging and laborious, limiting preventive actions. Approaches that do not rely on the TP itself being listed in databases like spectral similarity^28^ and molecular networking,^29^ using for example Global Natural Product Social Molecular Networking (GNPS),^30,31^ have been successfully applied to link precursors and TPs, thus facilitating structure elucidation, but still suffer from many false positive as well as false negative hits. While these approaches are promising, limitations remain, often leaving laborious manual structure elucidation as the final option. Improving the selectivity of these methods would represent a significant step toward high-throughput TP identification. Nonetheless, MS-based TP identification suffers from a lack of certainty unless a final confirmation with a reference standard is achieved (Level 1). Such standards are, however, rarely available for TPs.

## A Predictive Challenge?

Powerful prediction methods are needed to predict and prioritize a wide range of potential TPs and guide screening and other experimental approaches. Several tools for predicting TPs exist, some of which are integrated into analytical workflows such as patRoon.^32^ The majority focuses on predicting products of biotransformation as the key degradation pathway in most environments (e.g., enviPath,^33^ BioTransformer,^34^ PathPred,^35^ CATALOGIC,^36^ METEOR,^37^ etc.). There are fewer approaches targeted toward abiotic transformation processes such as ozonation (e.g., O3PPD^38^). In principle, such pathway prediction tools can be used to either directly predict major expected TPs to be considered during chemical management and/or to generate lists of expected TPs that can be used for analytical screening purposes. However, many well-known examples of persistent TPs, such as 1,2,4-triazole, or TFA (Figure 1b), indicate that these TPs may be formed after several transformation steps. Generalized biotransformation pathway prediction tools, while applicable to a wide range of chemicals, suffer from a lack of specificity in their prediction, which is exacerbated for later generations through the so-called combinatorial explosion. Predicted TP lists can become too long to provide sufficient discriminatory power, and the computational time becomes prohibitive after only a few generations. While methods have been developed to calculate the probability of predicted reactions and TPs, overall selectivity remains low, reaching 20–30% at best.^39^ Applying these tools for screening purposes therefore requires conscious optimization of the selectivity and sensitivity.

Improving tools for TP prediction requires more high-quality training data for structurally diverse chemicals. In that sense, the challenges faced with TP analysis and prediction are fundamentally similar: there is insufficient findable, accessible, interoperable, and reusable (FAIR) data available on TPs. The “Transformations” section in PubChem currently (2 April 2024) contains 6806 compounds, of which 5119 are TPs. This is only a tiny fraction of the entire PubChem database (118 million entries; 0.002% TPs), yet over double the data set used to train the prediction software BioTransformer.^34^ In principle, analysis generates data that help improve the predictive capabilities, while more reliable TP predictions reduce false positive and negative rates in TP screening, thus forming a positive feedback cycle. However, current limitations with TP identification and the lack of a sufficiently interoperable computer-readable structure format that allows for the expression of structural uncertainties make it challenging to efficiently annotate data with sufficient confidence to share in the public domain.

Beyond retraining existing algorithms, several ideas might be instrumental in focusing pathway prediction on potentially problematic TPs. First, pathway prediction could be combined with property prediction (i.e., ready degradability, half-lives, mobility, toxicity) to prioritize long lists of potential TPs. Second, if pathways are enumerated exhaustively, TPs that are not further degraded by any existing rule (and are not known metabolites of the central metabolism) can be identified. These are strong candidates for persistent TPs and should be given priority in analytical screening. Finally, knowledge of persistent substructures could be used to form a more targeted expansion of pathways to only continue branches that contain substructures likely to result in persistent TPs, e.g. C-CF_3_ in the case of TFA (Figure 1b).

## A Treatment Challenge?

Water treatment is one of the main mitigation strategies to remove chemicals from water and is expected to become even more important to cope with the increasing water scarcity caused by climate change and the resulting need to reuse water. While much attention is paid to the removal of organic micropollutants during water treatment, the formation of TPs during these processes is not studied to the same extent and is often neglected during the evaluation of the treatment efficiency. Aerobic and anaerobic microbial degradation, ozonation, and OH-radical-based technologies are used for treating wastewater and drinking water, causing a loss of primary biological activity for most micropollutants. However, these processes do not usually mineralize the organic constituents completely but rather lead to the formation of TPs. The commonly more mobile TPs are less removed than their precursors in subsequent treatment by activated carbon and can in some cases also be more toxic than the parent compound.^40−42^ Although this issue is widely known, the variety of potential TPs following oxidative treatment is poorly understood and remains underexplored.^43^ The same is true for byproducts of drinking water disinfection.^44,45^ In research on advanced oxidation processes there is a trend toward more selective oxidants (e.g., ^1^O_2_ or ferryl) with minimized scavenging by matrix components, such as dissolved organic matter (DOM), which may reduce full mineralization further,^46^ thus exacerbating TP formation. Biological post-treatment is frequently applied after disinfection or oxidation processes, as it was shown to efficiently remove some of the disinfection byproducts formed from DOM. However, more information is needed to assess its effectiveness for TP removal.^47^ Ecotoxicological testing can help assess the risk reduction of advanced water treatment options in a less biased manner.^48^ At the same time, structure-based insights,^49^ advanced kinetic modeling,^50^ and improved reactivity prediction tools (discussed above) may help to tailor oxidative treatment to reduce TP formation.

## A Regulatory Challenge?

TPs are comparatively well implemented in EU pesticide regulations,^51^ where their assessment is a key part of the risk assessment.^52^ This relatively advanced approach to pesticides in the EU, however, does not extend to chemicals policy in general, such as pharmaceuticals or industrial chemicals.

Regarding industrial chemicals, there are only very few cases in Europe’s REACH (Registration, Evaluation, Authorization, and Restriction of Chemicals) regulation, where there is an obligation to identify TPs. Even in these cases, TPs may be missed or excluded. Under REACH, registrants are required to carry out a PBT/vPvB (persistent, bioaccumulative, toxic, very persistent, and very bioaccumulative) assessment for all substances manufactured or imported above 10 tonnes per year, unless exemptions apply.^53^ If the substance is found to be readily or inherently biodegradable, it is assumed not to form any TPs relevant for a PBT/vPvB assessment.^53^ Ready biodegradability tests like OECD 301 can be passed based on, for example, 70% removal of dissolved organic carbon, or 60% of theoretical oxygen demand or CO_2_ production after 28 days^54^ to account for the test substances’ carbon being partially integrated into the biomass instead of mineralized. Consequently, low-carbon TPs such as TFA and 1,2,4-triazole may remain unrecognized in such tests even if formed in equimolar amounts (Figure 1b). Simulation tests in soil,^55^ water-sediment systems,^56^ and aquatic systems^57^ are to be conducted if a substance is found to not be readily or inherently biodegradable under REACH.

For the soil and water-sediment tests recommended in these guidelines, radioactive-labeled substances are used to track TPs. During these tests, REACH regulation 440/2008 requires the identification of TPs “accounting for ≥10% of the applied radioactivity or with constantly increasing concentrations unless reasonably justified”. However, unlike soil and water-sediment tests, the OECD 309^57^ test for water does not require the use of radioactive labeling and would therefore not identify TPs. Yet, OECD 309 is typically the default simulation test required unless it is technically not feasible for a given substance. Further, due to the complexity of simulation degradation tests, registrants can also use several weight-of-evidence-based approaches as part of the PBT/vPvB assessment to forego simulation tests entirely. Combined, this has meant that in practice very few simulation degradation tests have been conducted under REACH. A study from 2019 found simulation degradation test results for 292 out of the 22400 substances (1.3%) and 12960 (2.25%) unique identifiable organic substances registered under REACH.^58^ Thus, TPs are rarely assessed as part of this process.

For pharmaceuticals in Europe, there is even less obligation to identify TPs. However, it is recommended to follow guidelines similar to those of REACH as part of higher tier risk assessments.^59^ Looking toward major chemicals regulations in the two other large economic zones, the USA and China, TPs seem even less integrated, but progress is being made. A 2023 proposal^60^ for an update to the US EPA Toxic Substances Control Act^61^ foresees a stronger integration of TPs by addressing substances with “reasonably anticipated TPs” that cause serious acute or chronic effects or are PFAS or PBT substances. This neglects many mobile and persistent TPs, yet at the same time faces a major challenge to efficiently and reliably identify such TPs. China’s Ministry of Ecology and Environments Order 12, which went into force in 2020,^62^ requires degradability testing according to OECD 301 or 310^63^ (aerobic headspace test measuring CO_2_ evolution), and hence faces problems with TP detection during testing similar to those of REACH. The same issue also occurs in the most recent version of the United Nations Globally Harmonized System of Classification and Labeling of Chemicals (UN-GHS),^64^ which suggests OECD testing similar to that of REACH as part of Annex 9 on Guidance on Hazards to the Aquatic Environment; however, versions of the UN-GHS that recommend such testing have only been enacted among some UN member states.

Simulation degradation tests like OECD 307 and 308^55,56^ for soils and sediment-water systems, which allow for thorough TP identification, are rare in part because of their cost, complexity, and the regulatory challenge to enforce them. Therefore, weight-of-evidence-based persistency assessments are common, which do not provide any TP information. The main regulatory challenge can thus be linked to the lack of high-throughput persistence and TP assessment methods. Addressing the methodological challenges in persistence and TP assessment would, in the future, assist in addressing the regulatory ones, allowing chemicals regulation to be more ambitious with TPs and enforce their assessment more stringently. In the meantime, many persistent and toxic TPs of chemicals registered under REACH and other broad chemical regulations may remain unidentified and, thus, also unregulated.

## A Way Forward

Integrating TPs into chemicals assessment and management is an interdisciplinary and intersectoral endeavor for which three key advancements are (1) improved data exchange across sectors, (2) updated regulatory frameworks enabled by modernized testing regimes expediting TP identification and increased prediction capabilities, and (3) a broader view of transformation processes in the water cycle and product life cycles.

**Figure 2:**
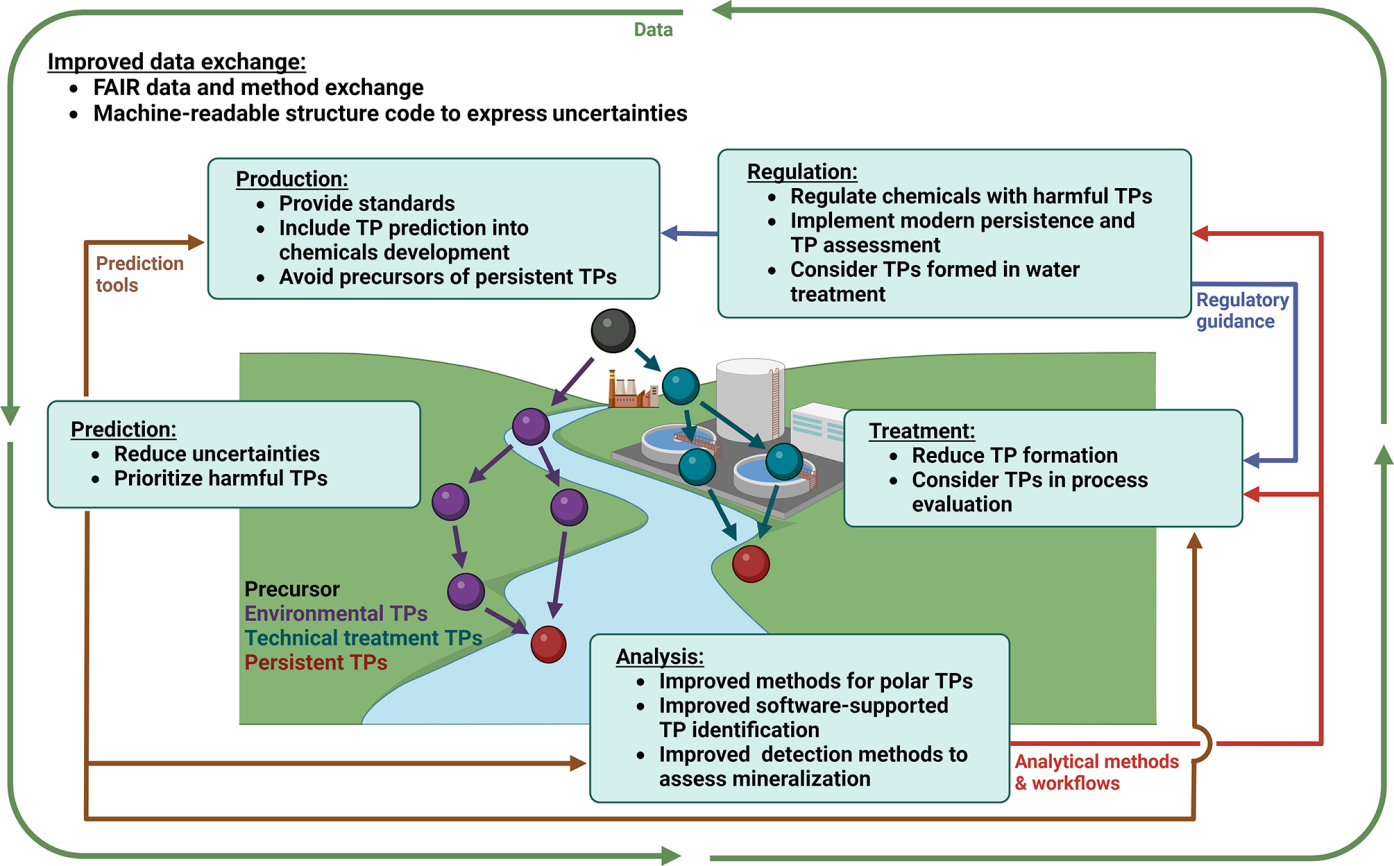
Proposed actions and interactions of researchers, industry, and regulation to facilitate a better integration of TPs into chemicals management.

Although the data available on TPs are constantly growing, neither can the rate of this growth keep pace with the ever-increasing number of precursor chemicals released and likely to be transformed in the environment^65^ nor is it sufficient to truly satisfy data-hungry machine-learning and artificial intelligence approaches.^66^ Thus, more data need to be generated and made available using more efficient high-throughput testing approaches, while existing data need to be exploited more effectively (e.g., through text mining^67^). To support this, FAIR templates, ontologies, and reporting standards^68,69^ ought to be implemented. An interoperable computer-readable structure code that allows the expression of structural uncertainties is essential for this, and a proposed InChI extension addressing exactly this use case has been submitted recently for consideration and future implementation by the InChI Trust.^70^ The implementation of such efforts may be significantly sped up if uploading TP information to a data repository becomes an explicit requirement by funding bodies, as established on a European level for monitoring data with the IPChem platform.^71^ REACH and similar regulations should be updated to make it mandatory for registrants to publicly share the results of their simulation studies and spectral data (e.g., mass spectra and NMR) of parent compounds and TPs as part of the registration process. Registrants may further be obliged to provide reference standards of both parent chemicals and their TPs to support environmental monitoring. Ultimately, the exchange of data among authorities, academia, and industry needs to be intensified (Figure 2) by reducing barriers and adopting a common data language and data sharing platforms.^72^

Expanded regulatory frameworks are required to oblige industry to identify more TPs. This includes a higher number of simulation tests and updated requirements that prevent low-molecular-weight TPs from being overlooked. To keep pace with the resulting substantial increase in required simulation tests, regulation must make the transition from the well-established, but now mostly over 20-year-old OECD tests, toward modernized high-throughput tests. Two such approaches are especially noteworthy in this context: Birch et al.^73^ assessed the persistence of a wide range of chemicals in low-concentration mixtures coupled with mass spectrometric detection. This high degree of parallelization allowed for high-throughput persistence testing but rendered it nearly impossible to assess mineralization and link detected TPs to their precursors within one experiment. Escher et al.^74^ proposed an experimental coupling of biotransformation experiments and high-throughput *in vitro* bioassays for P and *t* testing, which forgoes chemical analysis in favor of a “persistent toxicity” assessment. The advantage of this method is that it essentially treats parent compounds and TPs equally, since both persistent and toxic parent compounds or toxic TPs may cause this “persistent toxicity”. However, experimental challenges remain in identifying bioassays that adequately cover all relevant end points in higher organisms. Furthermore, solid phase extraction, which may result in the loss of the most polar TPs, is still a requirement. Both approaches seem promising and would benefit from methodological advancements in sample preparation to better enrich very polar substances, sensitive generic detection strategies that are inclusive of low-molecular-weight TPs, approaches that lower the costs associated with radiolabeling, and advanced software tools that help link TPs to precursors.

Increased testing capacities available through improved testing methods may also enable an extension of regulatory persistence and TP assessment beyond biotransformation. In such a case, transformation during oxidative water treatment and during a product life cycle seem most relevant for consideration. While the first will be realized for pesticides during drinking water production according to a new guidance document released by EFSA and ECHA,^75^ the latter has been shown to be relevant by the formation of the TP 6-PPDQ in tires.^4^ Such an extension may happen stepwise, first focusing on chemicals with known persistent or toxic moieties, and may be more broadly adopted if it proves to be a valuable addition to environmental protection.

## Implications

Effectively integrating TPs into chemicals management requires experimental and analytical progress, improved data sharing and prediction capabilities, integrated treatment technologies, and regulatory advancements that work hand in hand. Thus, an active discussion among authorities, academia, and industry is essential to achieve lasting progress. While such a substantial modernization of our chemical management paradigm is a long-term endeavor, it is an essential step toward more holistic environmental protection efforts. Thus, the foundation of such progress can and should be laid now. (I) The implementation of common data standards and a central repository for TPs may significantly move forward by being integrated in the recent “Proposal for a Regulation establishing a common data platform on chemicals”.^76^ (II) Increased requirements for sharing data and standards for chemicals by their producers can at first be focused on the chemicals themselves and major TPs identified in simulation studies. As chemical assessment evolves to include TPs more thoroughly, these requirements can be extended. (III) More collaboration between research and regulation is required to integrate modern methods for persistence and TP assessment into updated regulatory testing frameworks. Despite their usefulness, missing regulatory acceptance might delay the implementation of such methods substantially if no concerted effort is made to prove their applicability.

If the measures proposed herein are implemented, the increasing availability of high-quality TP data would reduce uncertainties in persistence and TP prediction, eventually reducing the reliance on simulation tests. Ultimately, these growing predictive capabilities may facilitate the effective consideration of TPs in early stages of chemical design and development, thus preventing chemicals with harmful TPs from ever reaching the market. Such an advancement would be a cornerstone for the development of safe and sustainable chemicals.

## Supporting Information Available

CRediT statement (PDF)
