# Supplementary material for: Should Transformation Products Change the Way We Manage Chemicals?
Source: Environ Sci Technol. 2024 Apr 24;58(18):7710–8. doi: 10.1021/acs.est.4c00125 (PMC11080041; doi:10.1021/acs.est.4c00125)
Supplement: Supplementary file 1 — es4c00125_si_001.pdf [file es4c00125_si_001.pdf]

CRediT statement

Daniel Zahn: Writing – Original Draft, Writing – Review & Editing, Visualization

Hans Peter Arp: Writing – Original Draft, Writing – Review & Editing

Kathrin Fenner: Writing – Original Draft, Writing – Review & Editing

Anett Georgi: Writing – Original Draft, Writing – Review & Editing

Jasmin Hafner: Writing – Review & Editing, Visualization

Sarah E. Hale: Writing – Original Draft, Writing – Review & Editing

Juliane Hollender: Writing – Review & Editing

Thomas Letzel: Writing – Review & Editing

Emma L. Schymanski: Writing – Original Draft, Writing – Review & Editing

Gabriel Sigmund: Writing – Original Draft, Writing – Review & Editing

Thorsten Reemtsma: Writing – Review & Editing
